# Non-human primate models and systems for gait and neurophysiological analysis

**DOI:** 10.3389/fnins.2023.1141567

**Published:** 2023-04-28

**Authors:** Fengyan Liang, Shanshan Yu, Siqi Pang, Xiao Wang, Jing Jie, Fei Gao, Zhenhua Song, Binbin Li, Wei-Hsin Liao, Ming Yin

**Affiliations:** ^1^Key Laboratory of Biomedical Engineering of Hainan Province, School of Biomedical Engineering, Hainan University, Haikou, China; ^2^Department of Rehabilitation Medicine, Affiliated Haikou Hospital of Xiangya Medical College, Central South University, Haikou, China; ^3^Shenzhen Institute of Advanced Technology, Chinese Academy of Sciences, Shenzhen, China; ^4^Department of Mechanical and Automation Engineering, The Chinese University of Hong Kong, Shatin, China

**Keywords:** non-human primate, gait, BCI, neurophysiological analysis, motor cortex

## Abstract

Brain–computer interfaces (BCIs) have garnered extensive interest and become a groundbreaking technology to restore movement, tactile sense, and communication in patients. Prior to their use in human subjects, clinical BCIs require rigorous validation and verification (V&V). Non-human primates (NHPs) are often considered the ultimate and widely used animal model for neuroscience studies, including BCIs V&V, due to their proximity to humans. This literature review summarizes 94 NHP gait analysis studies until 1 June, 2022, including seven BCI-oriented studies. Due to technological limitations, most of these studies used wired neural recordings to access electrophysiological data. However, wireless neural recording systems for NHPs enabled neuroscience research in humans, and many on NHP locomotion, while posing numerous technical challenges, such as signal quality, data throughout, working distance, size, and power constraint, that have yet to be overcome. Besides neurological data, motion capture (MoCap) systems are usually required in BCI and gait studies to capture locomotion kinematics. However, current studies have exclusively relied on image processing-based MoCap systems, which have insufficient accuracy (error: ≥4° and 9 mm). While the role of the motor cortex during locomotion is still unclear and worth further exploration, future BCI and gait studies require simultaneous, high-speed, accurate neurophysiological, and movement measures. Therefore, the infrared MoCap system which has high accuracy and speed, together with a high spatiotemporal resolution neural recording system, may expand the scope and improve the quality of the motor and neurophysiological analysis in NHPs.

## 1. Introduction

The past decades have seen the development of the lamprey (Grillner, 2003), cat (Clarac, 2008), rodent (McCrea and Rybak, 2008), sheep (Rizk et al., 2009), guinea pig (Sodagar et al., 2009), pig (Borton et al., 2011), and other animal models for locomotion. Specifically, non-human primates (NHPs) are critical in studying biomechanics, biodynamics, neurophysiology, pathophysiology, and evolution of humans, enabling the development of brain-computer interface (BCI) technologies.

NHPs have been widely selected as suitable subjects in neuroscience, especially BCI studies (Wessberg et al., 2000; Serruya et al., 2003; Griffith et al., 2008; Fitzsimmons et al., 2009; Foster et al., 2014; Yin et al., 2014) for several reasons. One is their unique phylogenetic proximity to humans (Lane, 2000). Thus, they offer a meaningful way to functionally evaluate neurotechnologies that have been designed for human subjects, enabling their effective translation to clinical settings (Badi et al., 2021). Second, NHPs and humans share similarities in functional brain structures. Third, NHPs share a similar diagonal interlimb synergy between the hindlimbs (legs) and forelimbs (arms) with humans, while nonprimate mammals have lateral sequence gait (Hildebrand, 1967; Courtine et al., 2005a,b). NHPs’ ability to walk bipedally like humans is of great interest in BCIs, as scientists seek to develop potential cures for gait deficits (Fitzsimmons et al., 2009). Last, NHPs can be trained to learn and perform more complicated tasksthan other animals, including reaching and grasping (Vargas-Irwin et al., 2010; Badi et al., 2021). In short, NHPs enable researchers to decode the relationship between intracortical activities and animal behaviors similar to humans (Zhang et al., 2012).

Neuroscience research, especially BCIs, the “brain-reading devices,” has been described as groundbreaking technology producing remarkable achievements. BCIs are promising to help restore movement, tactile sense, and communication in patients with paralysis (Hochberg et al., 2006, 2012; Collinger et al., 2013; Aflalo et al., 2015; Lebedev and Nicolelis, 2017; Willett et al., 2021; Drew, 2022). In 2004, BCI electrodes were embedded into the motor cortex of a human for the first time (Rapeaux and Constandinou, 2021). In 2021, a BCI that evoked tactile sensations and helped a patient with tetraplegia control prosthetic arms during reaching and grasping (Flesher et al., 2021), which is remarkable progress. In the same year, Willett et al. (2021) developed a BCI decoder to generate attempted handwriting, restoring communication in patients with paralysis. The study participant could type about 90 characters per minute, a speed comparable to the smartphone typing speeds of non-disabled individuals in the same age group. Behind all the achievements in humans, dozens of pilot neurophysiological experiments on NHPs were conducted for decades to decode how the brain senses and responds. In 1966, Evarts (1966) developed a technique for single-unit recording of pyramidal activities of tract neurons from awake, active NHPs for the first time. Georgopoulos et al. (1986) employed electrophysiological techniques to record single-neuron activities of NHPs during arm movements and found a strong correlation between the direction of reaching movement and a population of cortical neurons. In 2000, a population of cortical neurons in NHPs was processed to control a robotic arm in real-time (Wessberg et al., 2000).

Due to technological limitations, most NHP models for neural recordings are wired or tethered. The NHP is refined to a chair with its head and body fixed to protect the wire. Thus, only a few instructed arm movements can be studied. These models or paradigms are termed “head-fixed models” (Foster et al., 2014). However, arm movements are only a small subset of natural behaviors in NHPs. How the brain acts during other natural behaviors is still unclear and needs exploring.

**Figure 1:**
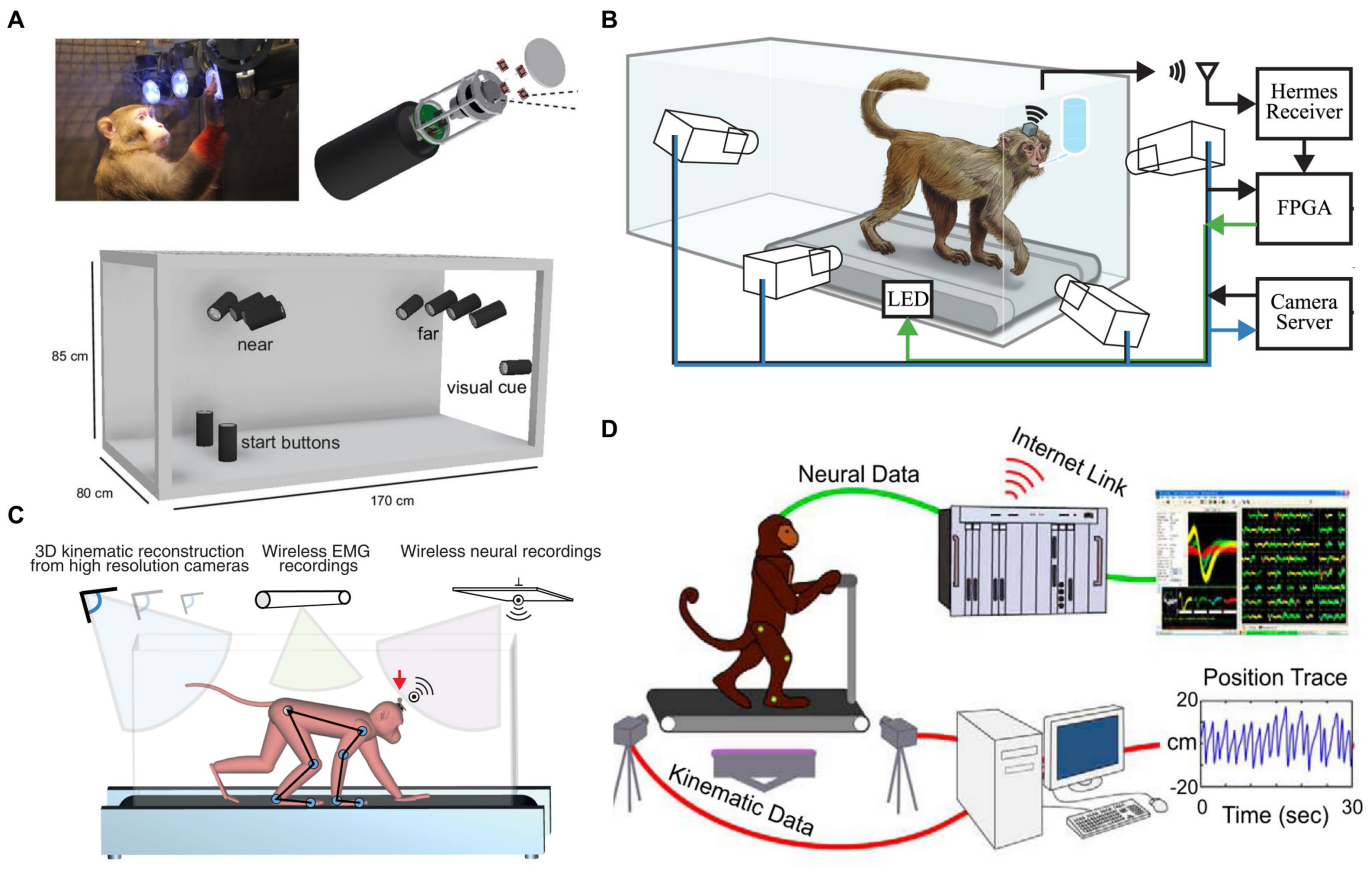
Freely-moving NHP models. **(A)** An unconstrained NHP in the Reach Cage [Reproduced with permission from Berger et al., 2020]. **(B)** Treadmill NHP model [Reproduced with permission from Foster et al., 2014]. **(C)** An unconstrained NHP on a treadmill [Reproduced with permission from Yin et al., 2014]. **(D)** Bipedal walking NHP model [Reproduced with permission from Lebedev and Nicolelis, 2017].

The advent of wireless neural recording systems for NHPs has expanded motor and neurophysiological analysis, enabling challenging setups requiring large or total freedom, such as locomotion. In 2004, Wise et al. (2004) pioneered a wireless implantable electronic interface to record cortical neural information. In 2007, Santhanam et al. (2007) presented a dual-channel neural recording system named HermesB for NHPs and humans. Since 2008, researchers, including our group, have continuously designed wireless neurosensors for full-spectrum neural recordings to expand brain research (Chestek et al., 2008; Harrison et al., 2008; Yin and Ghovanloo, 2009; Miranda et al., 2010; Rouse et al., 2011; Borton et al., 2013; Schwarz et al., 2014; Yin et al., 2014). Wireless neural recordings enable freely-moving NHP models or paradigms (e.g., Figure 1). A freely-moving monkey’s motion is recorded synchronously by a fast-speed MoCap system along with a high spatiotemporal resolution neural recording system. However, the first three studies to design freely-moving NHP models to analyze cortical neurons and locomotion comprehensively were all until 2014 (Foster et al., 2014; Schwarz et al., 2014; Yin et al., 2014).

Understanding the role of the motor cortex during locomotion has been a main research focus in the past decades. But it is unclear and needs to be further explored. There are two views on the neural control of movement (Shenoy et al., 2013). On the one hand, some believe that the motor cortex codes higher-level movement parameters, such as the position of the end-effector. On the other hand, the motor cortex was believed to code muscle action. In NHP arm movement, a population of cortical neurons is found to strongly correlate with movement direction (Georgopoulos et al., 1986). Wessberg et al. (2000) supported this finding and used a population of cortical neurons to control a prosthetic limb. After that, dozens of methods were proposed to decode or model the relationship between motor functions and recorded neural activity, including linear Wiener filters (Wessberg et al., 2000; Carmena et al., 2003), principal component analysis (PCA, Churchland et al., 2012), the Kalman filters (Wu et al., 2002; Li et al., 2009) and long short-term memory neural network (LSTM, a commonly-used recurrent neural networks, Tseng et al., 2019; Glaser et al., 2020). However, locomotion differs from arm movements regarding autonomy. It is found that the contributions of the motor cortex to locomotion and reaching movements are different (Xing et al., 2019). Drew (1988) found that the motor cortical neurons adjust the flexor muscle in cats during quadrupedal walking. In 2017, recent findings in a mouse model suggested that the role of motor cortical output in treadmill walking is significantly different from that in reaching movements (Miri et al., 2017). However, Yakovenko and Drew (2015) proposed that the motor cortex of cats plays a similar role in reaching and walking movements. These contradictory conclusions show a gap remains in understanding the role of the motor cortex during locomotion because of the need for more evidence from NHP models.

NHP models or paradigms designed in neuroscience studies require simultaneous, high-speed, accurate neurophysiological and movement measurements (Foster et al., 2014). There are already several methods or sensors proposed for human motion capture (MoCap), including ultrasound (Akaka and Houck, 1980), infrared beam arrays (Clarke et al., 1985), Doppler radar (Martin and Unwin, 1980), gyroscopes (Giansanti et al., 2005), and accelerometers (Van der Kruk and Reijne, 2018). Based on their physics principle, nowadays, commercially available MoCap systems can be categorized into five classes: optoelectronic systems (OS), image processing systems (IPS), electromagnetic systems (EMS), ultrasonic localization systems (ULS), and inertial sensory systems (ISS). These MoCap systems have been widely used in sports, neuroscience, computer vision, and robotics. The OS, including active marker systems and passive marker systems, are based on fixed cameras to capture the light reflected or emitted by the marker. The OS benefits from the semiconductor industry. It currently has the highest precision compared to other systems and is often regarded as the gold standard (e.g., Vicon or Optotrak) in the literature (Corazza et al., 2010; Galna et al., 2014; Van der Kruk and Reijne, 2018; Albert et al., 2020) for accurate quantitative movement-based analysis. However, using marker-based MoCap systems on NHP models faces challenges in making the animals comply with the marker setup; very often they remove or even swallow the marker.

This paper reviews NHP models and systems for gait and correlated neurophysiological research, focusing on MoCap and BCI neural recording systems for NHPs, to help researchers choose suitable systems for their experimental setup. An extensive literature review of peer-reviewed papers on NHP models and systems for gait and neurophysiological analysis is conducted. This review thoroughly evaluates various MoCap systems and neural recording devices used in NHP BCI and gait studies in the past. Based on their performances, useability, and data quality, the advantages and limitations of recent BCI and gait studies have been discussed. Finally, the challenges and directions of future NHP BCI and gait experimental setups are concluded.

## 2. Methods

A systematic review was conducted according to the preferred reporting items for systematic reviews and meta-analyses (PRISMA) guidelines (Page et al., 2021).

### 2.1. Search strategy

**Figure 2:**
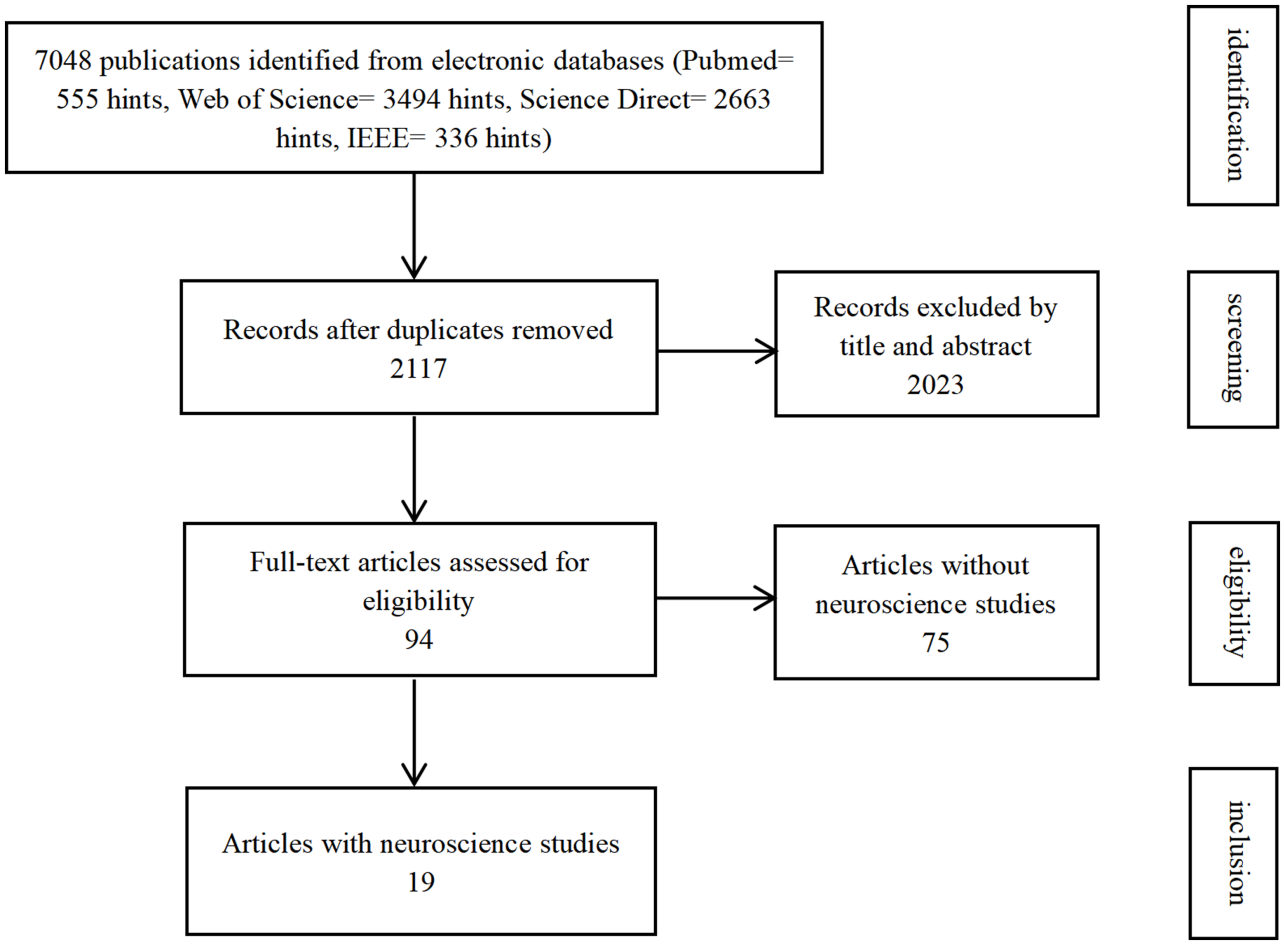
Flowchart of article selection.

A systematic literature search for neuroscientific studies of gait in NHP models was conducted in four main databases: Web of Science, Science Direct, PubMed, and IEEE, up until 1 June, 2022 (Figure 2). Some results searched in Google Scholar not covered by the four databases were also included as a supplement. The search strategy aimed to identify studies with NHP models for gait study, including the study that contains neuroscience. To exclude NHP gait studies that do not consider joint angles (e.g., only consider step numbers), we defined the search terms used as (*gait* OR *walking* OR *locomotion*) AND (*primates* OR *monkey* OR *macaque* OR *rhesus* OR *ape*) AND *angle*. Only publications in English were considered. The publication period investigated was from 2000 to June 2022.

First, the titles and abstracts of located papers were screened against the inclusion criteria. Then, the full texts of the papers were obtained and further screened for inclusion.

### 2.2. Inclusion criteria

This review includes NHP models and systems for gait and neurophysiological analysis in peer-reviewed papers. It focuses on the MoCap device/system and neurophysiological device, especially the BCI device.

### 2.3. Exclusion criteria

Book chapters, conference proceedings, review papers, and thesis works were excluded. The studies only considered NHP upper limb (not including locomotion) were also excluded.

### 2.4. Data management and statistical analysis

The characteristics of the included studies, such as the MoCap device, authors, other sensors, the neuroscience device, and the measurements, were tabulated. Subgroups were formed based on whether neural signals were studied in the paper.

## 3. Results

### 3.1. Publication overview

The database search initially returned 7,048 publications (Figure 2). We identified and excluded 4,931 duplicates. We assessed the remaining 2,117 publications for eligibility and ended up with 94 included studies. Nineteen studies were categorized into the subgroup as they also conducted neuroscience studies.

### 3.2. MoCap device

OS, IPS (including marker-based and markerless), footprint measurements, and observation and analysis from videos were employed in NHP studies (Table 1).

**Table 1:** MoCap systems in NHP studies.

	Price	Marker	Accuracy	Joint angles	Number of studies
OS	↑	√	↑↑	√	11
IPS-marker-based	↑	√	↑	√	44
IPS-markerless	↑		-	√	27
Observation and Analysis from Videos	-		↓		8
Footprints	↓		↓		4

A down arrow represents a lower extent, while an up arrow shows an increased extent from baseline (‘-’).

#### 3.2.1. OS

Infrared (IR)-based MoCap systems belonging to OS were used in a few studies (*n* = 11; 11.7%). Reflective or active markers were attached or painted on NHPs. Fixed infrared cameras emit the IR light and capture the light reflected by the markers to obtain the 3D locations of markers based on triangulation. Eleven studies employed three brands of IR-based MoCap systems: Qualisys (Blickhan et al., 2018, 2021; Ogihara et al., 2018; Oku et al., 2021), Optotrak (Xiang et al., 2007; Chen et al., 2009), and Vicon (Demes, 2011; Wei et al., 2016, 2018, 2019; Zhao et al., 2018). Qualisys and Vicon are passive marker systems, while Optotrak is an active marker system. IR light-emitting diodes (LEDs) emit IR light in the Optotrak system. How to put markers on NHPs is a challenging issue discussed in section 4.2.5.

#### 3.2.2. IPS

In IPS, video-captured films or photos are digitally analyzed. IPS has difficulty recognizing images in real-time and might require high-quality, high-speed cameras at a high cost (Van der Kruk and Reijne, 2018). IPS has marker-based tracking and markerless tracking. Note that both the marker-based IPS and the OS require markers, but marker-based IPS uses high-contrast markers detected by visible light, while OS employs reflective markers and works with infrared light. Moreover, markerless IPS usually tracks 2D features, such as corners or edges, and is convenient to use, especially for NHP models. For example, “DeepLabCut” is an up-to-date open-source tool for markerless pose estimation based on transfer learning. Its feasibility on humans, mice, macaque, and *Drosophila* was validated (Mathis et al., 2018; Mathis and Mathis, 2020; Labuguen et al., 2021). Specifically, Labuguen proposed an open-source dataset of Macaques in the wild for the training of DeepLabCut (Labuguen et al., 2021).

A large proportion of the NHP studies chose IPS (*n* = 71; 75.5%) because of its convenience. Among them, Frame DIAS (21 studies: e.g., Mori et al., 2006; Higurashi et al., 2010; Goto et al., 2021), Simi Motion (five studies: Fitzsimmons et al., 2009; Capogrosso et al., 2016; Vouga et al., 2017; Tseng et al., 2019; Xing et al., 2019), Peak Motus software (four studies: Courtine et al., 2005a,b; Larson and Demes, 2011; Demes and O’Neill, 2013), and Xcitex (seven studies: e.g., O’Neill et al., 2015; Thompson et al., 2018; Young and Chadwell, 2020) were commonly used (*n* = 37; 52.1%). The remaining 34 studies used self-written programs based on development platforms, such as MATLAB (Reghem et al., 2013, 2014; Pontzer et al., 2014; Dunham et al., 2018) and C++ (Peikon et al., 2009), or manually observed and analyzed videos or photos to obtain spatial–temporal parameters (Kimura, 2000; Nakano, 2002; Ma et al., 2016; Hu et al., 2021).

Commercial IPS supports both marker-based and markerless tracking. Among the 71 studies using IPS, 27 studies used markerless tracking. For example, Demes and O’Neill (2013) and Young and Chadwell (2020) chose prominent anatomical landmarks, such as the eyes and the base of the tails, to obtain normal spatial–temporal parameters, such as step length and walking speeds. Hirasaki et al. (2019) chose anatomical landmarks, such as wrist, elbow, hip, and knee joints, to get joint angles. The remaining 44 studies adopted marker-based tracking. Reflective or high-contrast paint markers in white or black on the shaved skin of NHPs were reported by all studies, except for Ogihara et al. (2010), which adopted reflective tape markers. Some studies also stressed that the paint was non-toxic (Goto and Nakano, 2018; O’Neill et al., 2018; Shitara et al., 2022b) or water-soluble (O’Neill et al., 2018), and the NHPs in the study were highly trained (Nakatsukasa et al., 2004).

The systems with X-rays also belong to IPS. Schmidt (2005, 2008) used an X-ray system to obtain the kinematics based on uniplanar cineradiography. However, the kinematics were captured only when the animals ran perpendicularly to the X-ray beam. Gait analysis was performed in specialized software (Unimark, by R. Voss, Tübingen, Germany).

#### 3.2.3. EMS, ULS, and ISS

None of the 94 included studies were reported to employ EMS, ULS, or ISS systems, maybe because of the inconvenience.

#### 3.2.4. Footprints measurement

Babu et al. (2007a,b, 2012) and Babu and Namasivayam (2008) did not employ cameras but footprint measurements to obtain spatial–temporal parameters. They painted non-toxic black ink on the feet of the NHPs. The animals left footprints on the white paper as they walked. But, this method is not reported in other NHP studies.

### 3.3. Accuracy of MoCap system

Different MoCap systems are with different accuracies (Table 2). The OS is believed to have the highest accuracy among other systems and is often regarded as the gold standard (e.g., Vicon or Optotrak) in the literature (Corazza et al., 2010; Galna et al., 2014; Van der Kruk and Reijne, 2018; Albert et al., 2020). For example, the Vicon system was reported to have an accuracy of 0.6 mm (Spörri et al., 2016) and 0.77 mm (listed in Table 2, Monnet et al., 2014). The OS outperformed IPS (Scaramuzza and Fraundorfer, 2011).

**Table 2:** The reported accuracy of MoCap systems.

References	System	Type	Cameras	Range/area (m)	Accuracy (°)	Accuracy (mm)
Dorociak and Cuddeford (1995)	Vicon 370	OS	5	2 × 1 m	0.2 at knee 0.05 at ankle	
Monnet et al. (2014)	Vicon T-40	OS	10	1.1 × 1 m		0.77
Spörri et al. (2016)	Vicon MX 13	OS	24	41.2 × 20 m		0.6
Ceseracciu et al. (2014)	SMART-D BTS	IPS-markerless	8	7 × 5 m	11.75 at knee 17.62 at hip	
Perrott et al. (2017)	Organic Motion	IPS-markerless	14	lab room	15.9 at knee	
Ruß (2016)	Simi Shape	IPS-markerless	8	lab room	6.4 in hip flexion 9.2 in hip rotation 20.6 in elbow	
Foster et al. (2014)	home-made	IPS-markerless	8	cage		26.1 at wrist
Nakamura et al. (2016)	home-made	IPS-markerless	4	cage	>35 at head	>40 in limbs
Hanley et al. (2017)	Simi Motion	IPS-marker-based	NA	NA	4 in knee	
Klous et al. (2010)	Simi Motion	IPS-marker-based	5	35 × 15 m		11 in x-axis 9 in y-axis 13 in z-axis

The feasibility and accuracy of markerless IPS still need to be improved due to the lack of proof in the literature (Ceseracciu et al., 2014). Ceseracciu et al. (2014) reported that markerless IPS has a root mean square error (RMSE) of 11.75°, 18.35% of the range of motion in the knee angle (in the sagittal plane), and an RMSE of 17.62°, 44.66% of the range of motion in the hip angle, compared with the gold standard in gait analysis. Similarly, significant differences in the knee angle (15.9°) during squats were reported by Perrott et al. (2017). Moreover, insufficient accuracy of the markerless system in the transverse plane is believed to hinder its application. For example, the Simi markerless system (Simi Reality Motion Systems GmbH, Unterschleißheim, Germany) was reported to have a standard deviation of 6.4° (±2.9°) in the sagittal plane hip angle, 9.2° (±3.8°) in the transverse plane hip angle and 20.6° (±28.3°) in elbow rotation (Ruß, 2016). The calculation error may be due to the camera’s optical distortion, the system’s processing errors, and skin artifacts (the system assumes that the model is rigid, whereas the skin is flexible during movement; Winiarski, 2003). Up to now, the Simi system’s accuracy or the validity of measurement is not yet validated by high-quality peer-reviewed studies.

Home-made IPS still has poor kinematics measurement. Foster et al. (2014) designed a markerless IPS with 8 video cameras for freely-moving NHPs. The estimated distance error for the wrist point is 26.1 mm. Nakamura et al. (2016) used four depth cameras (Kinect V1, Microsoft Corp., Redmond, WA, USA) to build a markerless system for NHPs. The estimated errors are reported as 4–14 cm for the position of different limbs and 35–43° for head rotation (listed in Table 2).

Marker-based IPS is usually more accurate than markerless IPS (Van der Kruk and Reijne, 2018). Simi system also supports marker-based tracking. The photogrammetric errors of the marker-based Simi system were reported as 11, 9, and 13 mm in the x, y, and z directions, respectively (Klous et al., 2010). An RMSE of 4° (±1°) in knee angle between Simi marker-based tracking and Qualisys (the gold standard) was reported during one gait cycle in race walking (Hanley et al., 2017). However, these accuracies are significantly lower than OS’ (listed in Table 2). Six studies adopted video observation and analysis but could only obtain the basic spatiotemporal parameters with poor accuracy. Four studies used footprint measurements in NHP’s locomotor analysis. This method is convenient and low-cost, but the accuracy and gait parameters obtained are not enough for the requirement of neuroscience studies.

### 3.4. Measurement of gait parameters

Parameters involved in gait studies provide scales to quantify animal behaviors, including spatiotemporal parameters, joint angles, ground reaction force (GRF), dynamics, neurophysiological measurements, and others.

#### 3.4.1. Spatiotemporal parameters

Commonly measured spatiotemporal parameters (SP) are stride time, step time, stance time, gait velocity, cadence, stride symmetry, and the number of steps. Most NHP studies measured spatiotemporal parameters (*n* = 92; 97.9%). Conversely, Ma et al. (2016) proposed a self-made score to quantify hindlimb locomotion in the NHP after spinal cord injury (SCI). The score was graded based on the manual video analysis. Revuelta et al. (2012) determined freezing of gait (FOG), a critical feature in Parkinson’s disease, in an NHP Parkinson’s model by reviewing the videos.

#### 3.4.2. Joint angles

Of the 94 NHP studies, 68 (72.3%) measured joint angles, such as those of the hip, knee, and ankle angles. Joint angle is a critical feature in NHPs. For example, Demes (2011) researched three-dimensional kinematics of capuchin monkey bipedalism by putting reflective markers over the major joints of the animals and employing Peak Motus software (PEAK Performance Technologies Inc., Centennial, CO, USA) to calculate several angles (listed in Table 3 with definitions). Joint angles can quantify gait ability in SCI NHPs. Capogrosso et al. (2016) proposed a brain–spinal interface to alleviate gait disabilities in NHPs after SCI. The MoCap system, Simi, used 4–6 video cameras at 100 Hz along with reflected white paint markers on shaved skin to measure 7 landmarks on the animal’s right side. 3D spatial coordinates of the markers were obtained using the Simi motion tracking software and then the joint angles were computed accordingly. The increased knee angle in the paretic leg of NHPs showed the recovery of locomotion and demonstrated the feasibility of brain–spinal interface on NHPs and its potential application for humans.

**Table 3:** Joint angles measured in Demes (2011).

Angles	Definition
Trunk pitch angles	Sagittal plane angles of hip-shoulder with vertical
Trunk tilt angles	Frontal plane angles of hip-shoulder with vertical
Hip abduction	Angle between trunk and thigh in frontal plane
Hip angles	Vector angle between trunk and thigh
Knee angles	Vector angle between shank and thigh
Ankle angles	Vector angle between shank and foot
Foot angles	Angle of ankle-head of 5th metatarsal with horizontal
Protraction at touchdown	Angle of hip-5th metatarsal with horizontal at touchdown
Retraction at lift off	Angle of hip-5th metatarsal with horizontal at lift off
Hind limb angular excursion	Sum of protraction and retraction

#### 3.4.3. GRF and dynamics

One common way to obtain the dynamics in gait analysis is to measure the GRF during locomotion. Seventeen NHP studies adopted force plate(s) to measure GRF in the NHP experiments. Force plates can be combined with MoCap systems, such as Qualisys (Qualisys AB, Gothenburg, Sweden. Blickhan et al., 2018, 2021; Ogihara et al., 2018; Oku et al., 2021), Frame-DIAS (DKH, Tokyo, Japan. Higurashi et al., 2010; Ogihara et al., 2010, 2012; Shimada et al., 2017), and Xcitex (Xcitex Inc., Cambridge, MA, USA. Thompson et al., 2018; Young and Chadwell, 2020) for the calculation of joint dynamics by the system software. Some studies measured GRF not to obtain dynamics but rather the center of mass. Demes et al. (2015) used force plates to analyze the center of mass mechanics of an NHP during bipedal walking. Hanna et al. (2015) analyzed the center of mass during load-carrying locomotion in NHPs.

### 3.5. Neurophysiological measurements

Neurophysiological measurements in NHP studies, such as electromyogram (EMG) and cortical neural recordings, are discussed in section 4.

### 3.6. Other measurements

NHP gait studies often involve gait parameter measurements and neurophysiological analysis; however, a few studies measure other locomotion-related signals. Nakatsukasa et al. (2004, 2006) employed an infrared gas analyzer in NHP studies to measure and analyze the energy consumption during bipedal and quadrupedal walking by measuring CO_2_ production.

## 4. Discussion

In the discussion, we focus on NHP studies involving neuroscience research (19 studies, listed in Table 4), while those that do not measure any neurophysiological data were not considered. NHP neuroscience studies include intracortical neural recordings (i.e., BCI), EMG, motor-evoked potential (MEP), and somatosensory-evoked potential (SSEP).

**Table 4:** NHP neuroscience studies.

References	Device	Method/Software	Gait Measurement	Biodevice	Wireless	Tethered
Berger et al. (2020)	IPS	DeepLabCut	SP	BCI	√	
Capogrosso et al. (2016)	IPS	Simi motion	Joint angles, SP	BCI	√	
Courellis et al. (2019)	IPS	Mean shift algorithm	SP	BCI		√
Fitzsimmons et al. (2009)	IPS	Simi motion	Joint angles, SP	BCI		√
Foster et al. (2014)	IPS	Computer vision algorithm	Joint angles, SP	BCI	√	
Goetz et al. (2012)	IPS	Dartfish ProSuite	Joint angles, SP	Surface EMG, BCI		√
Hazama and Tamura (2019)	IPS	Matlab	SP	BCI		
Hu et al. (2021)	Videos	Observation and Analysis	SP	Implantable EMG		√
Ma et al. (2016)	Videos	Observation and Analysis	a self-made scale, MEP, SSEP	BCI		√
Nakano (2002)	Videos	Observation and Analysis	SP	Surface EMG		√
Peikon et al. (2009)	IPS	Self-written software in GNU C++	Joint angles, SP	BCI		√
Schwarz et al. (2014)	IPS	Computer vision algorithm	SP	BCI	√	
Shitara et al. (2022a,b)	IPS	Frame-DIAS	Joint angles, SP	Implantable EMG	√	
Tseng et al. (2019)	IPS	Simi motion	Joint angles, SP	BCI		√
Vouga et al. (2017)	IPS	Simi motion	Joint angles, SP	BCI		√
Wei et al. (2019)	OS	Vicon software	Joint angles, SP	Surface EMG		√
Xing et al. (2019)	IPS	Simi motion	Joint angles, SP	BCI	√	
Yin et al. (2014)	IPS	Simi motion	Joint angles, SP	BCI, Implantable EMG	√	

### 4.1. Biodevice

NHP studies of intracortical neural recordings or BCI were based on home-made (e.g., HermesB) or commercial neural recording systems (e.g., Blackrock Microsystems, Salt Lake City, UT, USA; FHC Inc., Bowdoin, ME, USA). EMG studies employed implantable electrodes (Yin et al., 2014; Hu et al., 2021; Shitara et al., 2022b) or surface EMG sensors (Nakano, 2002; Goetz et al., 2012; Wei et al., 2019). Ma et al. (2016) adopted MEP and SSEP to evaluate spinal cord function electrophysiologically in NHP models.

### 4.2. BCI

There are 12 NHP BCI and gait studies from eight research teams (listed in Table 5). The BCIs in two of the 12 studies were implanted in the hippocampus, while BCIs in other studies were all in the motor cortex. Three studies by researchers at Duke University (Fitzsimmons et al., 2009; Peikon et al., 2009; Tseng et al., 2019) were based on the same tethered setup or paradigm. The NHPs walked bipedally on a treadmill with two hands fixed. Before the experiments, the NHPs were tattooed, and fluorescent, non-toxic markers were applied. A tethered multichannel neural acquisition system (Plexon, Inc., Dallas, TX, USA) was connected to headstages, enabling the simultaneous recording of neural activity and capture of movement during the experiments. But in 2014, Schwarz et al. (2014) from Duke University designed a wireless large-scale recording system to enable tethered and wireless freely-moving NHP models.

**Table 5:** NHP BCI and gait studies.

Studies	University	Implant area	Bipedal	Quadrupedal	Device	Method/Software	Marker	How to fix markers	Wireless	Tethered
Berger et al. (2020)	University of Goettingen	Motor cortex		√	IPS	DeepLabCut			√	
Courellis et al. (2019)	University of California	Hippocampus		√	IPS	Mean shift algorithm	√	2 LEDs placed on the marmoset’s head cap		√
Foster et al. (2014)	Stanford University	Motor cortex		√	IPS	Computer vision algorithm			√	
Goetz et al. (2012)	Grenoble Institut Neurosciences	Motor cortex	√		IPS	Dartfish ProSuite				√
Hazama and Tamura (2019)	University of Toyama	Hippocampus		√	IPS	Matlab	√	Two light bulbs fixed at head cap		√
Fitzsimmons et al. (2009)	Duke University	Motor cortex	√		IPS	Simi motion	√	Tattooed, White fluorescent makeup (Kryolan)		√
Peikon et al. (2009)	Duke University	Motor cortex	√		IPS	Self-written software in GNU C++	√	Tattooed, White fluorescent makeup (Kryolan)		√
Schwarz et al. (2014)	Duke University	Motor cortex	√	√	IPS	Self-written program			√	
Tseng et al. (2019)	Duke University	Motor cortex	√		IPS	Simi motion	√	Tattooed, White fluorescent makeup (Kryolan)		√
Capogrosso et al. (2016)	EPFL	Motor cortex		√	IPS	Simi motion	√	Shaved, Reflective white paint	√	
Xing et al. (2019)	Brown University	Motor cortex	√	√	IPS	Simi motion	√	Shaved, Reflective white paint	√	
Yin et al. (2014)	Brown University	Motor cortex		√	IPS	Simi motion	√	Shaved, Reflective white paint	√	

None of the 12 BCI and gait studies adopted OS but IPS. Because the OS faces challenges in making the animals comply with the marker setup; very often, they just remove or even swallow the marker. A better solution is needed to use OS, the gold standard with the highest accuracy in NHP’s BCI and gait studies.

#### 4.2.1. Wireless

Transmitting neural data wirelessly enables a wide range of natural behavior studies with full-body movements, including arm movements and locomotion (listed in Table 6). Compared with wired neural recording, wireless rexording does not need to tether the NHP, thus the NHP is freely-moving. Moreover, the training complexity in freely-moving NHPs is lower than in tethered ones.

**Table 6:** Comparison of wired and wireless neural recording systems and models.

References	Neural recording	Tethered	Freely-moving	Training complexity	Arm movements	Bipedal	Quadrupedal
Fitzsimmons et al. (2009)	Wired	√		-	√	Unnatural	
Foster et al. (2014), Schwarz et al. (2014), Yin et al. (2014)	Wireless		√	↓	√	√	√

A down arrow represents a lower extent from baseline (‘-’).

A few studies (*n* = 6) performed wireless neural recordings (i.e., there is no wire between the BCI headstages and recording systems). For example, wireless modules designed by Yin et al. (2014; Figure 1C) enabled neural signal transmission to external receivers in the studies conducted at EPFL and Brown University (Yin et al., 2014; Capogrosso et al., 2016; Xing et al., 2019). Stanford University (Figure 1B; Foster et al., 2014) used home-designed HermesD and HermesE systems for the wireless transmission of neural data. Both Capogrosso et al. (2016) and Berger et al. (2020; Figure 1A) used a commercial neural recording system (CerePlex W, Blackrock Microsystems, Salt Lake City, UT, USA). The system offers 96 ch wideband neural recording with 16 b resolution, 30 kSps sampling rate, weighs only 33.5 g with battery, and measures 32.5 × 32.5 × 21 mm. It has a wireless transmission range of 3 m and 2 m for freely moving animals in rich multipath fading environments. But the system still requires improvement in coverage distance, battery life, and channel counts.

#### 4.2.2. Tethered

Studies undertaken at Duke University (Fitzsimmons et al., 2009; Peikon et al., 2009; Tseng et al., 2019), University of California (Courellis et al., 2019), Grenoble Institute Neurosciences (Goetz et al., 2012), and University of Toyama (Hazama and Tamura, 2019) were based on tethered NHP models, while other BCI studies enabled freely-moving NHPs (listed in Table 5). Fitzsimmons et al. (2009; Figure 1D) developed an NHP bipedal walking paradigm. Monkeys were trained to walk on the treadmill with arms holding a bar on the treadmill. A wired BCI system was used to obtain neural recordings from the M1 area. They reported that neurons in M1 modulate the firing rate to the timing of gait cycles. But monkeys’ arms holding a bar breaks the interlimb coordination between forelimbs and hindlimbs during nature gait. Conversely, freely-moving NHP models are promising to help understand how the brain changes with the environment and how to control prosthetic devices in response to neuron signals.

#### 4.2.3. Bipedal or quadrupedal

Due to the limitation of their wired recording systems, three studies at Duke University forced the NHPs to walk bipedally on a treadmill with two hands holding a bar. Therefore, only bipedal gait was analyzed. Moreover, fixing forelimbs may affect the nature of bipedal gait. By contrast, freely-moving NHP models in other studies enabled the study of bipedal and quadrupedal gait. Schwarz et al. (2014) and Xing et al. (2019) obtained bipedal, quadrupedal gait, and intracortical neural data.

#### 4.2.4. MoCap system

All 12 NHP BCI and gait studies used the IPS. However, the accuracy of IPS is not comparable with OS, as discussed in section 3.3. Goetz et al. (2012), Foster et al. (2014), and Berger et al. (2020) used markerless IPS systems, whose accuracy is outperformed by marker-based IPS. Simi motion is a marker-based IPS system commonly used in five BCI and gait studies. However, as mentioned in section 3.3, the accuracy of Simi is not yet validated by high-quality peer-reviewed studies. Thus, the reported error (4–20°) does not meet the requirement of high-speed, precise kinematics and neural decoding. However, Simi can be used in BCI and gait studies that do not need accurate kinematics due to its convenience. For example, Capogrosso et al. (2016) used Simi to compare the gait of the NHP SCI model before and after the intervention (brain–spinal interface). Joint angles, step height, and foot trajectory of the NHP were obtained and compared.

Up to now, no NHP BCI and gait studies have used OS, which has the highest accuracy as the gold standard (Corazza et al., 2010; Galna et al., 2014; Van der Kruk and Reijne, 2018; Albert et al., 2020). The OS requires the placement of rigid reflective markers on NHPs. Thus, attaching reflective markers on NHPs to enable OS in BCI and gait studies becomes a key issue.

#### 4.2.5. Marker attachment

In the six NHP BCI and gait studies that used marker-based IPS systems, reflective white paint or white fluorescent makeup was commonly used as markers on the NHPs. The reflective white paint provides high contrast to other colors on NHPs and thus can be easily captured by the IPS.

**Figure 3:**
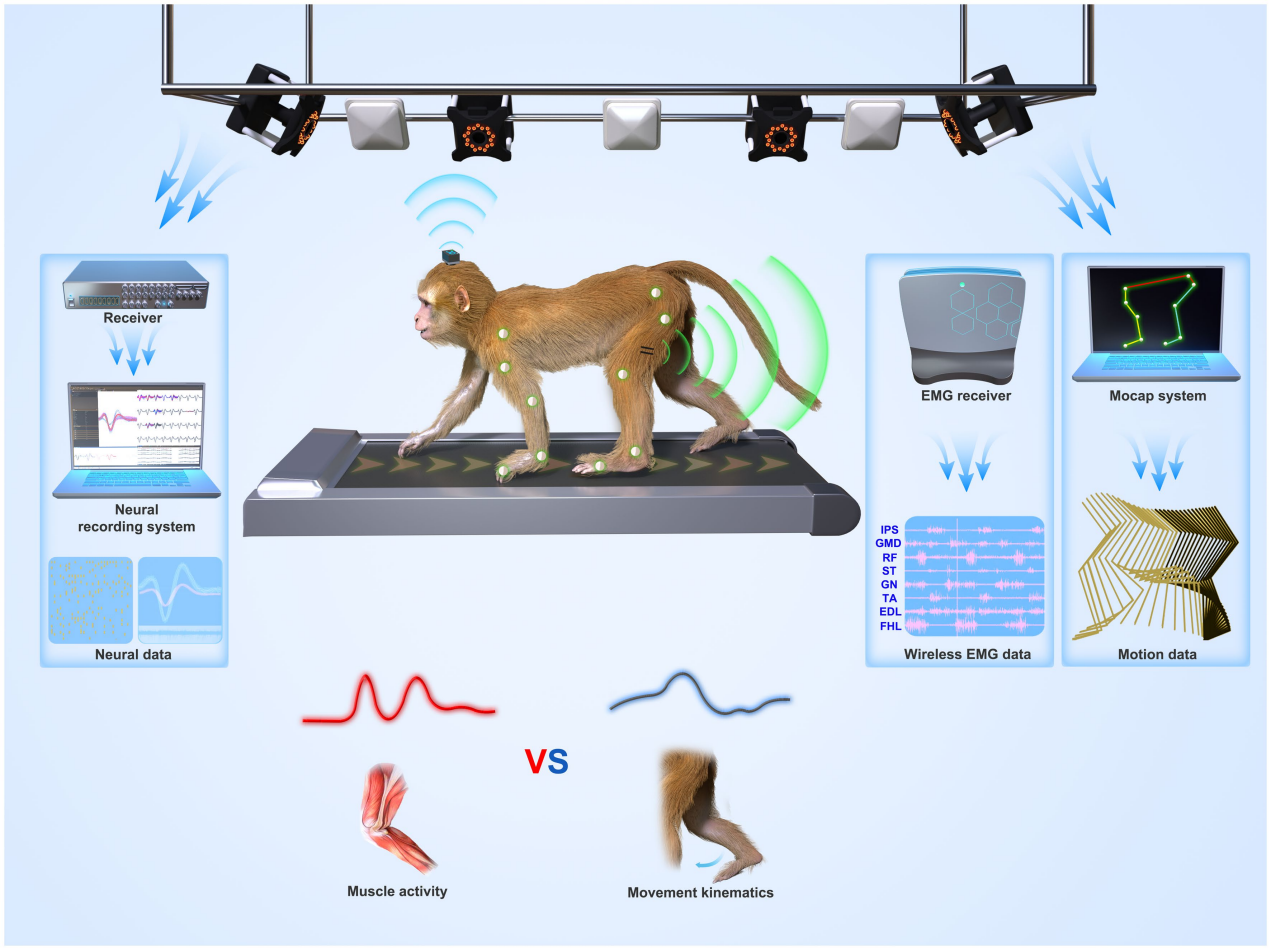
System overview of a suggested freely-moving (untethered) NHP model or paradigm. Wireless neural, EMG, and motion data from a monkey were recorded synchronously. The paradigm can be used to understand whether the motor cortex codes muscle activities (lower left panel) or movement parameters (lower right panel) during locomotion.

Although no NHP BCI and gait studies used OS, there is still some experience from 11 NHP gait studies that used OS (introduced in 3.2.1). To attach the rigid reflective markers to NHPs, one way (four studies) is to make NHPs walk on a treadmill with two forelimbs fixed (Wei et al., 2016, 2018, 2019; Zhao et al., 2018). This method disables the study of quadrupedal gait. Another way (seven studies) is to attach the markers with straps or tapes. For example, Velcro straps, double-sided tape, and children’s leggings were used by Blickhan et al. (2018, 2021), Ogihara et al. (2018), and Oku et al. (2021), whereas Xiang et al. (2007) and Cohen et al. (2009) adopted Optotrak active OS. In the experiments, IR LEDs (rigid bodies, or “markers”) were fixed on tapes and then wrapped around the head, chest, wrists, and ankles. Optotrak system requires training the NHPs not to eat or grasp the tape or the markers, and this issue may influence the experiment. Moreover, fixing forelimbs may affect the nature of bipedal gait. Both approaches have their weaknesses. A better solution is needed to use OS in BCI and gait studies. Finding replacements for markers, such as self-made surface markers, may be a solution. Recently, our group is setting up an OS-based freely-moving NHP model (Figure 3) with a high accuracy and fast speed MoCap system (Mars 4H, Nokov Science and Technology Co., Ltd., Beijing, China) along with a high spatiotemporal resolution neural recording system and wireless EMG system, to increase the accuracy of kinematics and thus expand motor and neurophysiological analysis in NHPs.

#### 4.2.6. Findings or contribution

With the MoCap systems, 12 NHP BCI and gait studies have different findings or contributions. Capogrosso et al. (2016) proposed a brain-spinal interface to alleviate gait disabilities in NHPs after SCI. The increased knee angle obtained by the MoCap system in the paretic leg showed the recovery of locomotion and stressed the potential of the brain-spinal interface for humans. Three studies undertaken at Duke University established tethered NHP bipedal models that enabled online decoding of 3D kinematics from intracortical neural signals. Subsequently, a better computational decoder, the LSTM, was proposed to replace the Kalman filter (Tseng et al., 2019) because LSTM units have physiological features like neuron populations, such as neuronal dynamics, and directional tuning. In 2014, three studies of different universities all built freely-moving NHP models using self-developed wireless neural recording systems (Foster et al., 2014; Schwarz et al., 2014; Yin et al., 2014). Following those studies, Xing et al. (2019) simultaneously recorded bipedal and quadrupedal kinematics with intracortical data. They found that it was possible to decode kinematics from a limited number of neurons just as well as or better than from the neural population (18–80 neurons). However, the limited accuracy of the MoCap system (Simi, reported error: 4–20°) may influence the robustness of their findings. Berger et al. (2020) designed a free-moving NHP model based on a commercial-available wireless recording system (Blackrock Microsystems, Salt Lake City, UT, USA) and a markerless motion capture system to study goal-directed movements, including walk-and-reach.

Two studies analyzed the role of the hippocampus’s place cells in locomotion. Hazama and Tamura (2019) found that NHP’s place cells are more sensitive to locomotion velocity than direction. Courellis et al. (2019) reported that the correlation between the activities of place cells and θ oscillations in NHP is remarkably different from rodents.

## 5. Current challenges and opportunities

### 5.1. The role of the motor cortex in gait generation remains unsolved

The role of the motor cortex in NHP arm movement has been studied for a long time. Whether the motor cortex codes muscle activities or higher-level movement parameters such as limb trajectory is an open question (Figure 3). In other words, does the cortical activity correlate with the muscle EMG or with movement kinematics such as position and velocity? In 1986, a population of cortical neurons was found to have strong correlations with movement direction (Georgopoulos et al., 1986). But, Cisek (2006) argued that the motor system does not describe the motion but produces it. Moreover, evidence from cats suggested a direct contribution of the motor cortex to leg muscle adjustment during gait modifications (Drew and Marigold, 2015). However, studies from another view sought to describe the firing rate of motor neuron populations as the function of various kinematic parameters such as hand or joint kinematics, target locations, and the estimated endpoint error (Reimer and Hatsopoulos, 2009; Wang et al., 2010; Pearce and Moran, 2012). Locomotion movements are different from arm movements regarding autonomy. There is still a lack of consensus on the role of the motor cortex during locomotion due to the lack of related studies.

Due to technological limitations of neural recording systems, only three papers (Foster et al., 2014; Yin et al., 2014; Xing et al., 2019) studied the role of the motor cortex during NHP’s quadrupedal locomotion and only one of them (Xing et al., 2019) analyzed the role of the motor cortex regarding kinematics, not just spatial–temporal parameters. Our group (Yin et al., 2014) reported the reproducible cyclic ensemble modulation of NHP motor cortex across the entire gait cycle during natural locomotion. Foster et al. (2014) found that the modulation of NHP’s motor cortex correlates well with wrist speed during locomotion. However, neither Yin nor Foster gave insights into gait kinematics such as joint angles; their gait parameters are limited to spatial–temporal parameters. Xing et al. (2019) found that it was possible to decode hindlimb kinematics and gait phase from limited neurons just as well or as accurately as from the neural population (18–80 neurons). It is the first demonstration that the motor cortex can robustly decode kinematics and gait during NHP quadrupedal locomotion. But the limited accuracy of the MoCap system (Simi, reported error: 4–20°) used in their research may influence the robustness of their findings. Moreover, their findings lack evidence, considering they are the only study analyzing gait kinematics and neural recordings simultaneously during NHP quadrupedal locomotion.

Therefore, since the advent of wireless neural recording technology has paved the way for freely-moving BCI and gait studies in NHPs, one of the future study directions is to clarify the role of the motor cortex during locomotion, especially the accurate correlation or model between motor cortex activities and gait kinematics by using high spatiotemporal resolution neural recording along with high accuracy and fast speed MoCap systems.

### 5.2. Decoding or modeling methods in BCI and gait studies

Wessberg et al. (2000) employed a population of cortical neurons to control a prosthetic limb. After that, dozens of methods were proposed to decode or model the relationship between the motor of upper limbs and recorded neural activities in NHP’s arm movement tasks, including linear Wiener Filters (Wessberg et al., 2000; Carmena et al., 2003), PCA (Churchland et al., 2012), Kalman Filters (Wu et al., 2002; Li et al., 2009), and LSTM (Tseng et al., 2019; Glaser et al., 2020). Wessberg et al. (2000) employed a linear filter and an artificial neuron network (ANN) to decode neural population signals to estimate real-time three-dimensional hand movements and control a robotic device for the first time. No significant differences were reported between these two decoders. Churchland used PCA, the popular dimensionality reduction technique, to extract essential features at the single-neuron level from neuron populations during arm reaching in NHPs. Tseng et al. (2019) LSTM was reported to outperform Kalman Filter, the state-of-the-art method in decoding arm movements from neuron populations.

However, since the role of the motor cortex during locomotion is unclear, whether the decoding methods for arm movement can model neuron populations in the motor cortex and gait kinematics is unknown. Xing et al. (2019) successfully employed Poisson Linear Dynamical System (PLDS) to reconstruct limb kinematics from neuron populations in the motor cortex. He suggested that the state-of-art RNN may outperform PLDS. He also found a difference between the contributions or functions of the motor cortex to locomotion and reaching movements. Except for Xing’s work, no other studies tried to model the neuron populations in the motor cortex and gait kinematics. Moreover, the limited accuracy of the MoCap system (Simi, reported error: 4–20°) used in Xing’s study influenced the robustness of his conclusions.

Therefore, future studies need to: testify if motor neuron populations and gait kinematics can be modeled using previous decoding methods for arm movements such as PLDS and PCA; testify if the contributions or functions of the motor cortex in bipedal locomotion, quadrupedal locomotion, and arm movements are different; try to find an optimal decoding method to fully extract the gait kinematics from cortical neuron populations and give insights for the generation mechanism of locomotion.

### 5.3. Wireless neural recording device

While wireless neural recording devices can free animals and offer unprecedented opportunities for recording signals under untethered natural behavior, building a feasible wireless system for NHP BCI and gait research faces many technical challenges. The most critical ones include wireless coverage and quality, data throughput, size, and power consumption. It is even more challenging since all the above specs are cross related. Usually, NHP gait experimental space needs to be at least 1 m × 1 m × 1 m (Schwarz et al., 2014), with some extending up to a few cubic meters (CerePlex W, Blackrock Microsystems, Salt Lake City, UT, USA). Depending on the wireless recording system channel counts, a high data rate comes with high channel counts. For example, 96 channels with a 30kSps sample rate (to record high-frequency neural action potentials) and 16-bit resolution will produce roughly 50Mbps data throughput. With such a high data rate, wireless transmission with a few meters of reliable coverage and low power (in the 10s of mW range) is tough, especially when the applications require high-fidelity wireless communication with a minimum bit-error rate. Plus, a minimum bit-error rate often requires complicated power-hungry error detection and correction coding in wireless transceivers. With the increased system complexity and power dissipation, the systems need to use larger batteries and produce more heat. The former increase the size and weight of the device, hinder usability, and potentially biases the animal behavior; the latter could harm the animal. Therefore, low-power, reliable, high data rate short distance wireless communication is key to a successful wireless recording device for NHPs BCI and gait application.

Previously, Schwarz et al. (2014) built a custom wireless neural recording system to record high-frequency neural data from a maximum of 1792 channels. For every 128 channels, the data was transmitted through a commercial ISM band radio (Nordic nRF24L01+) with 1.33 Mbps data throughput after intensive data compression. To transmit all 1792 channels, they used 16 ISM radios. And the overall transceiver power reached 2 mW/ch, leading to 256 mW per 128 ch and over 4 W for the whole 1792 channel system. As a result, the battery and size of the entire system are fairly large (roughly 10 cm × 10 cm × 10 cm). Additionally, the original 16-bit sample data was shortened to 8-bit, and lookup table compression was used to free up extra bits further to leverage the limited 1.33 Mbps ISM radio data throughput. Foster et al. (2014) presented a wireless neural recording system (Hermes D and E) for a freely-moving monkey treadmill model. They used custom design ASIC and device to implement the system, which achieved 24 Mbps transmission of 128 ch data at 12.5 kSps/ch sampling rate. But the wireless power consumption is still high (125 mW) and the sampling rate is relatively low for Aps recordings (usually around 30 kSps/ch is the industrial standard). The entire device was enclosed in a 38 × 38 × 51 mm aluminum enclosure. Though its power dissipation, size, sampling rate, and data throughput still require further improvement, the system is small and light-weighted enough for NHPs applications. Yin et al. (2014) proposed a wireless system for locomotion analysis using custom wireless recording ASICs and devices, the power consumption of the system has been limited to around 82.5 mW for 100 channels, with the wireless communication part to be around 4 mW/15 mW (low and high output power modes). The size of the device was 52 × 44 × 30 mm and it can function over 48 h from a single 1.2 Ah one-half AA Li-SOCL2 primary battery. SIMO wireless communication technology was used to improve signal fidelity. Although the system has quite a few improvements in size, weight, power dissipation, and signal fidelity, it still experienced issues in wireless coverage distance and potential signal loss due to the multipath fading effect from the animals’ freely moving. Animals moving causes received signal strength to vary over a large scale. Capogrosso et al. (2016), Berger et al. (2020), and Silvernagel et al. (2021) all used CerePlex W system from Blackrock Microsystem. The system offers 96 ch wideband neural recording with 16 b resolution, 30 kSps sampling rate, weighs only 33.5 g with battery, and measures 32.5 × 32.5 × 21 mm. It has a wireless transmission range of 3 m and 2 m for freely-moving animals in rich multipath fading environments. It can operate for 3.5 h continuously without recharging the battery, which is mostly sufficient for applications like NHPs BCI and gait research. But the system still requires improvement in coverage distance, battery life, and channel counts.

Overall, wireless systems attract many researchers’ attention in the field due to the ability to free the animals and enable NHP research requiring natural behavior in an untethered setup, which provides more genuine un-bias neural data. However, because of technical challenges, such an ideal wireless recording system is yet to come and awaits further engineering.

## 6. Conclusion

Our understanding of the motor cortex remains incomplete (Shenoy et al., 2013), and further research is needed to understand its role in locomotion and neural control in NHP. This knowledge is essential to provide insights into the generation mechanism of walking in humans, thus giving implications for the control design of capable, accurate neural prostheses and biped robots (Shenoy et al., 2013) and for the rehabilitation of gait disorders such as stroke. NHP has been commonly selected as a suitable subject in neuroscience studies. This review summarizes 94 NHP studies with gait analysis, including 12 studies with BCI and gait studies. While wired neural recording systems have been used to acquire electrophysiological data in NHPs, the advent of wireless neural recording systems has expanded the scope of neuroscience research on freely-moving NHPs, enabling challenging experimental setups requiring large or total freedom, such as locomotion. Our group has continuously designed wireless neurosensors for full-spectrum neural recordings (Yin and Ghovanloo, 2008, 2009, 2011; Yin et al., 2013, 2014). However, with the harsh requirements for a complete NHP locomotion research setup, it was in 2014 that the first three studies came out with designs of freely-moving NHP models to comprehensively analyze cortical neurons and locomotion (Foster et al., 2014; Schwarz et al., 2014; Yin et al., 2014).

Limited research (Foster et al., 2014; Yin et al., 2014; Xing et al., 2019) has been conducted on the role of the motor cortex during NHP quadrupedal locomotion. The study of Xing et al.’s (2019) is the only study that attempts to decode hindlimb kinematics from a limited number of neurons in NHP quadrupedal locomotion; the low accuracy MoCap system used in his study compromised the robustness of his conclusions. Thus, the role of the motor cortex in gait generation, including bipedal and quadrupedal gait, remains unsolved, and the suitable methods to decode or model the neuron populations in the motor cortex and gait kinematics are also unclear. Future studies need to (1) clarify the role of the motor cortex during locomotion, especially the accurate correlation or model between motor cortex activities and gait kinematics; (2) quantify and compare the contributions or functions of the motor cortex in bipedal locomotion, quadrupedal locomotion, and arm movements; (3) investigate if motor neuron populations and gait kinematics can be modeled using previous decoding methods for arm movements such as PLDS and PCA; (4) testify if the state-of-art RNN may outperform previous decoding methods (as suggested by Xing et al.); (5) identify an optimal decoding method to fully extract the gait kinematics from cortical neuron populations for freely-moving bipedal and quadrupedal gait; (6) explore contributions from other cortical areas (such as posterior parietal cortex) to the control of locomotion.

The advent of wireless neural recording systems for NHPs enabled freely-moving NHP models and neuroscience research on NHP locomotion. However, current MoCap systems in BCI and gait studies are based on image processing and lack accuracy (error: ≥4° and 9 mm). Some home-made IPS systems used in NHP models have been reported even higher measurement errors (26.1 mm and > 40 mm). To address this issue, future BCI and gait studies require simultaneous, high-speed, and accurate measures of neural and movement data. Neuroscience studies may consider using a commercial infrared MoCap system (OS) that is considered the gold standard with the highest accuracy (Figure 3). However, the challenge is how to place markers on NHPs’ bodies as they may remove or even eat the marker. This review discussed the methods used in the literature and suggested that self-made surface markers may be a solution. Additionally, CerePlex W system (Blackrock Microsystem, Salt Lake City, UT, USA, Figure 3) is the most frequently-used and validated commercial wireless neural recording system in current BCI studies. But, there is still room for improvement in coverage distance, battery life, and channel counts. Home-made wireless neural recording systems should also consider signal quality, data throughput, working distance, size, and power constraints. Moreover, other measurement systems such as wireless EMG systems (Figure 3) and force plates can be combined with commercial MoCap systems to obtain EMG and gait dynamics, such as joint force and torque, to enhance neuroscience studies. Currently, our group is working to set up an OS-based NHP model and improve neural recording systems for NHP neuroscience studies.

## Author contributions

FL and SY contributed to the conception, investigation, and original draft of the study. SP provided methodological input. XW and JJ provided scientific input and contributed to the manuscript writing. ZS and BL contributed to the manuscript editing. FL wrote the first draft of the manuscript. FG, W-HL, and MY supervised the whole project and reviewed the manuscript. All authors contributed to the article and approved the submitted version.

## Funding

This research was supported by the Key R&D Project of Hainan Province (Grant Nos. ZDYF2022SHFZ302, ZDYF2022SHFZ275, and ZDYF2021SHFZ083), the High-level Talent Project of Natural Science Foundation of Hainan Province (Grant Nos. 322RC560 and 821RC532), the National Natural Science Foundation of China (No. 32160204), the Major Science and Technology Projects of Hainan Province (Grant No. ZDKJ2021032), and Hainan Province Clinical Medical Center (No: 0202067).

## Conflict of interest

The authors declare that the research was conducted in the absence of any commercial or financial relationships that could be construed as a potential conflict of interest.

## Publisher’s note

All claims expressed in this article are solely those of the authors and do not necessarily represent those of their affiliated organizations, or those of the publisher, the editors and the reviewers. Any product that may be evaluated in this article, or claim that may be made by its manufacturer, is not guaranteed or endorsed by the publisher.
